# Low-Rank Linear Dynamical Systems for Motor Imagery EEG

**DOI:** 10.1155/2016/2637603

**Published:** 2016-12-21

**Authors:** Wenchang Zhang, Fuchun Sun, Chuanqi Tan, Shaobo Liu

**Affiliations:** ^1^The State Key Laboratory of Intelligent Technology and Systems, Computer Science and Technology School, Tsinghua University, FIT Building, Beijing 100084, China; ^2^Institute of Medical Equipment, Wandong Road, Hedong District, Tianjin, China

## Abstract

The common spatial pattern (CSP) and other spatiospectral feature extraction methods have become the most effective and successful approaches to solve the problem of motor imagery electroencephalography (MI-EEG) pattern recognition from multichannel neural activity in recent years. However, these methods need a lot of preprocessing and postprocessing such as filtering, demean, and spatiospectral feature fusion, which influence the classification accuracy easily. In this paper, we utilize linear dynamical systems (LDSs) for EEG signals feature extraction and classification. LDSs model has lots of advantages such as simultaneous spatial and temporal feature matrix generation, free of preprocessing or postprocessing, and low cost. Furthermore, a low-rank matrix decomposition approach is introduced to get rid of noise and resting state component in order to improve the robustness of the system. Then, we propose a low-rank LDSs algorithm to decompose feature subspace of LDSs on finite Grassmannian and obtain a better performance. Extensive experiments are carried out on public dataset from “BCI Competition III Dataset IVa” and “BCI Competition IV Database 2a.” The results show that our proposed three methods yield higher accuracies compared with prevailing approaches such as CSP and CSSP.

## 1. Introduction

With the development of the simpler brain rhythm sampling technique and powerful low-cost computer equipment over the past two decades, a noninvasive brain-computer interface (BCI) called electroencephalography (EEG) has attracted more and more attention than other BCIs such as magnetoencephalography (MEG), functional magnetic resonance imaging (fMRI), and near infrared spectroscopy (NIRS). Among various EEG signals, certain neurophysiological patterns can be recognized to determine the user's intentions such as visual evoked potentials (VEPs), P300 evoked potentials, slow cortical potentials (SCPs), and sensorimotor rhythms. EEG brings hope to patients with amyotrophic lateral sclerosis, brainstem stroke, and spinal cord injury [1]. Motor imagery (MI), which is known as the mental rehearsal of a motor act without real body movement execution, represents a new approach to access the motor system for rehabilitation at all stages of the stroke recovery. People with severe motor disabilities can use EEG-BCI to realize the communication and control and even to restore their motor disabilities [2, 3]. Therefore, an increasing number of researchers are working on MI-BCI for stroke patient rehabilitation [4, 5].

MI-BCI concentrates on sensorimotor *μ*- or *β*-rhythms that has the phenomenon known as event-related synchronization (ERS) or event-related desynchronization (ERD). However, the MI pattern recognition is still a challenge due to the low signal-to-noise ratio, highly subject-specific data, and low processing speed. For these reasons, more and more digital signal processing (DSP) methods and machine learning algorithms are applied to the MI-BCI analysis. Unlike static signals such as images and semantics, the EEG signals are dynamic that lie in a spatiotemporal feature space. Thus a large variety of feature extraction algorithms are proposed, including power spectral density (PSD) values [6, 7], autoregressive (AR) parameters [8, 9], and time-frequency features [10]. For MI-BCI pattern recognition, there are mainly three types of methods: autoregressive components (AR) [11], wavelet transform (WT) [12, 13], and CSP [14, 15]. Because of effectiveness and simplicity in extracting spatial features, CSP becomes one of the most popular and successful solutions for MI-BCI analysis according to the winners' methods analysis of “BCI Competition III Dataset IVa” [16, 17] and “BCI Competition IV Database 2a” [18, 19]. Therefore, many researchers devote theirselves to improving the original CSP method for better performances, such as common spatiospectral pattern (CSSP) [20], common sparse spectral spatial pattern (CSSSP) [21], subband common spatial pattern (SBCSP) [22], filter bank common spatial pattern (FBCSP) [23], wavelet common spatial pattern (WCSP) [24], and separable common spatiospectral patterns (SCSSP) [25]. Most of these improved CSP methods fuse spectral and spatial characteristics in the spatiospectral feature space and finally achieve success by comparison experiments.

Despite its effectiveness in extracting features of MI-BCI, CSP needs a lot of preprocessing and postprocessing such as filtering, demean, and spatiospectral feature fusion, which influence the classification accuracy easily. In this paper, we utilize linear dynamical systems (LDSs) for processing EEG signals in MI-BCI. Although LDSs succeed in the field of control, to the best of our knowledge, this model has barely been tried in the feature extraction of EEG analysis so far. Compared with CSP method, LDSs have the following advantages: first, LDS can simultaneously generate spatiospectral dual-feature matrix; second, there is no need to preprocess or postprocess signals, and the raw data can be directly fed into the model; third, it is easy to use and of low cost; last, the extracted features from the LDS are much more effective for classification.

Furthermore, we apply low-rank matrix decomposition approaches [26–28] that have the ability to learn representational matrix even in presence of corrupted data. The noise of the data can be get rid of, hence to improve the robustness. However, there are two ways for the EEG low-rank decomposition. One aims at the EEG raw data; the other aims at features extracted from LDSs, which is proposed by us and called low-rank LDSs (LR-LDSs).

This paper mainly has the following contributions. (1) We utilize LDSs for MI-EEG feature extraction to solve the MI pattern recognition problem. (2) Low-rank matrix decomposition method is applied to improve the robustness for the raw data analysis. (3) We propose LR-LDSs on finite Grassmannian feature space. (4) Plenty of comparison experiments demonstrate the effectiveness of these approaches.

The rest of this paper is organized as follows.  Section 2 provides LDSs model to realize the feature extraction of EEG signals.  Section 3 presents low-rank matrix decomposition method for the EEG raw data analysis.  Section 4 introduces LR-LDSs method. Then, the proper classification algorithm is explained in Section 5.  Section 6 compares the three proposed methods (LDSs, LR+CSP, and LR-LDSs) with other state-of-the-art algorithms in different databases. Finally, the summary and conclusion are presented in Section 7.

## 2. LDSs Modeling

LDSs, also known as linear Gaussian state-space models, have been used successfully in modeling and controlling dynamical systems. In recent few years, more and more problems extending to computer vision [29, 30], speech recognition [31], and tactile perception [32] have been solved by LDSs model. EEG signals are sequences of brain electron sampling that have typical dynamic textures. We present the features of EEG dynamic textures by LDSs modeling and apply machine learning (ML) algorithms to capture the essence of dynamic textures for feature extraction and classification.

Let {*Y*(*t*)}_*t*=1,…,*τ*_, *Y*(*t*) ∈ *R* ^*m*^ be a sequence of *τ* EEG signal sample at each instant of time *t*. If there is a set of *n* spatial filters *φ*_*α*_ : *R* → *R* ^*m*^, *α* = 1,…, *n*, we have *x*(*t*) = ∑_*i*=1_^*k*^ *A*_*i*_ *x*(*t* − *i*) + *Bv*(*t*) with *A*_*i*_ ∈ *R* ^*n*×*n*^, *B* ∈ *R* ^*n*×*n*_*v*_^, independent and identically distributed realization item *v*(*t*) ∈ *R* ^*n*_*v*_^ and suppose that sequence of observed variables *Y*(*t*) can be represented approximately by function of dimensional hidden state *x*(*t*), *y*(*t*) = *φ*(*x*(*t*)) + *ω*(*t*), where *ω*(*t*) ∈ *R* ^*m*^ is an independent and identically distributed sequence drawn from a known distribution resulting in a positive measured sequence. We redefine the hidden state of *x*(*t*) to be ${\left[\begin{array}{cccc} {x\left(t\right)}^{T} & {x\left(t-1\right)}^{T} & \cdots & {x\left(t-k\right)}^{T} \end{array}\right]}^{T}$ and consider a linear dynamic system as an autoregressive moving average process without firm input distribution:

(1) xt+1=Axt+Bvt,yt=φxt+ωt,x0=x0

with *v*(*t*), *φ*(*x*(*t*)) distribution unknown, however.

In order to solve the above problem, we can regard it as a white and zero-mean Gaussian noise linear dynamical system and propose a simplified and closed-form solution:

(2) xt+1=Axt+Bvtvt~N0,Q,yt=Cxt+ωt+y−ωt~N0,R,x0=x0,

where *A* ∈ *R* ^*n*×*n*^ is the transition matrix that describes the dynamics property, *C* ∈ *R* ^*m*×*n*^ is the measurement matrix that describes the spatial appearance, $\overset{-}{y}\in R^{m}$ is the mean of *y*(*t*), and *v*(*t*) and *ω*(*t*) are noise components. We should estimate the model parameters *A*, *C*, *Q*, *R* from the measurements *y*(*t*),…, *y*(*τ*) and transform them into the maximum-likelihood solution:

(3) A^τ,C^τ,Q^τ,R^τ=argmaxA,C,Q,R⁡ py1,…,yτ,

and, however, optimal solutions of this problem bring computational complexity.

We apply matrix decomposition to simplify the computation by the closed-form solution. The singular value decomposition (SVD) solution is the best estimate of *C* in Frobenius function:

(4) C^τ,X^τ=argminC,X WτFsubject  to Yτ=CXτ+Wτ; CTC=I.

Let *Y* = *U*Σ*V* ^*T*^, and we get the parameter estimation of $\hat{C}$, $\hat{X}$:

(5) C^=U,X^=ΣVT,

where $\hat{X}=[X\left(1\right),X\left(2\right),\ldots ,X\left(\tau \right)]$. $\hat{A}$ can be determined by Frobenius:

(6) $$\begin{array}{c} \hat{A}\left(\;\tau \;\right)=\underset{A}{\mathrm{argmin}}{\left\|\;X_{2}\left(\;\tau \;\right)-AX_{1}\left(\;\tau -1\;\right)\;\right\|}_{F}, \end{array}$$

where *X*_2_(*τ*) = [*X*(2), *X*(3),…, *X*(*τ*)]. So the solution is in closed-form using the state estimated

(7) A^τ=X:,2X:,3⋯X:,τ∗X:,1X:,2⋯X:,τ−1†,

where † denotes matrix pseudoinverse.

We can obtain the result [*A*, *C*], a couple of spatiotemporal feature matrix. The MATLAB program of LDSs can be found in Supplementary Material algorithm 1 available online at http://dx.doi.org/10.1155/2016/2637603.

## 3. Low-Rank Matrix Decomposition

EEG signals have poor quality because they are usually recorded by electrodes placed on the scalp in a noninvasive manner that has to cross the scalp, skull, and many other layers. Therefore, they are moreover severely affected by background noise generated either inside the brain or externally over the scalp. Low-rank (LR) matrix decomposition can often capture the global information by reconstructing the top few singular values and the corresponding singular vectors. This method is widely applied in the field of image denoising and face recognition (FR). Concretely, low-rank (LR) matrix recovery seeks to decompose a data matrix *X* into *A* + *E*, where *A* is a low-rank matrix and *E* is the associated sparse error. Candès et al. [33] propose to relax the original problem into the following tractable formulation:

(8) minA,E A∗+αE1s.t. X=A+E,

where the nuclear norm ‖*A*‖_*∗*_ (the sum of the singular values) approximates the rank of *A* and the *l*_1_-norm ‖*E*‖_1_ is sparse constraint.

Then, Zhang and Li [34] decompose each image into common component, condition component, and a sparse residual. Siyahjani et al. [35] introduce the invariant components to the sparse representation and low-rank matrix decomposition approaches and successfully apply to solve computer vision problems. They add orthogonal constraint to assume that invariant and variant components are linear independent. Therefore, we decompose EEG signals as a combination of three components: resting state component, motor imagery component represented by low-rank matrix, and a sparse residual. However, in practice, it needs some digital signal processing (DSP), that is, wavelet transform or discrete Fourier transform before decomposition. Particularly, raw time-domain signals without any preprocessing are not suitable for low-rank matrix decomposition directly. The training dataset *X* can be decomposed by *X*≔*A* + *B* + *E*, where *A* ∈ *R* ^*m*×*n*^ is a low-rank matrix and collects event-related EEG signal components, *B* ∈ *R* ^*m*×*n*^ approximates invariant and denotes resting state signal components that are sampled by subjects without any motor imagery, and *E* ∈ *R* ^*m*×*n*^ is the matrix of sparse noise. Therefore, training dataset can be decomposed as the following formulation:

(9) $$\begin{array}{c} X≔A+B+E. \end{array}$$

On ideal condition, each sampling channel of subject's brain EEG signals in resting state is similar. In other words, sum of each different *A* raw is minimum. *B* should add common constraint as the following formulation:

(10) $$\begin{array}{c} \underset{i\neq j}{\sum }{\left\|\;B_{i}-B_{j}\;\right\|}_{F}^{2}. \end{array}$$

We propose optimization problem formulation as

(11) minA,B,E A∗+αE1+β∑i≠jBi−BjF2s.t. X=A+B+E.

Then, augmented Lagrange multiplier (ALM) [36] method is utilized to solve the above problem. The augmented Lagrangian function *L*(*A*, *B*, *E*, *λ*) is given by

(12) LA,B,E,λ=A∗+αE1+β∑i≠jBi−BjF2+λ,X−A−B−E+μ2X−A−B−EF2,

where *μ* is a positive scalar and *λ* is a Lagrange multiplier matrix. We employ an inexact ALM (IALM) method described in Algorithm 1 to solve this problem, where *J*(*X*) = max⁡(lansvd⁡(*X*), *α* ^−1^‖*X*‖_*F*_) in the initialization *λ*_0_ = *X*/*J*(*X*) and lansvd(·) computes the largest singular value.

When low-rank matrix *A* denoting event-related EEG signal components are generated, we can utilize some feature extraction methods such as CSP and CSSP to classify MI-BCI. In other words, low-rank matrix decomposition method in this section can be considered as a preprocessing part before feature extraction and classification.

## 4. LR-LDSs on Finite Grassmannian

Beginning at an initial state *x*1, the expected observation sequence generated by a time-invariant model *M* = (*A*, *C*) is obtained as *E*[*y*_1_, *y*_2_, *y*_1_,…] = [*C* ^*T*^, (*CA*)^*T*^, (*CA* ^2^)^*T*^, …]^*T*^ *x*1 that lies in the column space of the extended observability matrix given by *O*_*∞*_^*T*^ = [*C* ^*T*^, (*CA*)^*T*^, (*CA* ^2^)^*T*^, …]^*T*^ ∈ *R* ^*∞*×*n*^. LDSs can apply the extended observability subspace *𝒪* as descriptor, but it is hard to calculate. Turaga et al. [37, 38] approximate the extended observability by taking the *L*-order observability matrix; that is, *O*(*n*, *L*) = [*C* ^*T*^, (*CA*)^*T*^, …,(*CA* ^*L*−1^)^*T*^]^*T*^. In this way, an LDS model can be alternately identified as an *n*-dimensional subspace of *R* ^*Lm*^.

Given a database of EEG, we can estimate LDSs model and calculate the finite observability matrix that span subspace as a point on the Riemannian manifold. Then, based on low-rank and sparse matrix decomposition, observability matrix *O* can be decomposed into *D* + *E* as the following formulation:

(13) minD,E D∗+αE1s.t. O=D+E,

where *D* is a low-rank matrix and *E* is the associated sparse error.

The inexact ALM method can be also used to solve the optimization problem like Algorithm 1. The output *D* represents low-rank descriptor for LDSs and can be employed for the classification of EEG trails.

## 5. Classification Algorithm

We extract features by the above LDSs model and get two feature matrices *A* and *C*. Unfortunately, *A* and *C* have different modal properties and dimensionalities. So they cannot be represented directly by a feature vector. Riemannian geometry metric for the space of LDSs is hard to determine and needs to satisfy several constraints. Common classifiers such as Nearest Neighbors (NNs), Linear Discriminant Analysis (LDA), and Support Vector Machines (SVM) cannot classify features in matrix form. The feature matrix must be mapped to vector space. We use Martin Distance [39, 40], which is based on the principal angles between two subspaces of the extended observability matrices, as kernel to present distance of different LDS feature matrix. It can be defined as

(14) $$\begin{array}{c} D^{2}\left(\;\Theta_{a},\Theta_{b}\;\right)=-2\overset{n}{\underset{i=1}{\sum }}\log\lambda_{i}, \end{array}$$

where Θ_*a*_ = {*C*_*a*_, *A*_*a*_}, Θ_*b*_ = {*C*_*b*_, *A*_*b*_}. *λ*_*i*_ is the eigenvalue solving as the following equation:

(15) 0OabOabT0xy=λOaa00Obbxys.t.  xTOaax=1,  yTObby=1,

where the extended observability matrices *𝒪*_*a*_ = [*C*_*a*_^*T*^, *A*_*a*_^*T*^ *C*_*a*_^*T*^,…, (*A*_*a*_^*T*^)^*n*^ *C*_*a*_^*T*^], *𝒪*_*b*_ = [*C*_*b*_^*T*^, *A*_*b*_^*T*^ *C*_*b*_^*T*^,…, (*A*_*b*_^*T*^)^*n*^ *C*_*b*_^*T*^], *𝒪*_*ab*_ = (*𝒪*_*a*_)^*T*^ *𝒪*_*b*_. Algorithm 2 in Supplementary Material presents Martin Distance function programed by MATLAB.

We can classify EEG signals by comparing Martin Distance between training data and testing data. Nearest two samples mean that they may be of the same class. So the forecast label and predict accuracy can be calculated. Algorithm 3 in Supplementary Material is the classification method of KNN.

Considering LR-LDSs methods generating *A* on Finite Grassmannian, unlike two feature matrices (*A*, *C*) by LDSs, Euclidean Distance and Mahalanobis Distance can describe the distance between two feature spaces of EEG trails after LR-LDS. They are simple, efficient, and common for measuring distance between two points. In order to improve the accuracy of classification, we can also employ metric learning methods using the label information to learn a new metric or pseudometric such as neighborhood components analysis and large margin nearest neighbor.

## 6. Experimental Evaluation

From the above sections, we propose three methods for EEG pattern recognition: LDSs, LR+CSP, and LR-LDSs. Two datasets of motor imagery EEG including BCI Competition III Dataset IVa and BCI Competition IV Database 2a are used to evaluate our three methods compared with other state-of-the-art algorithms such as CSP and CSSP. All experiments are carried out with MATLAB on Intel Core i7, 2.90-GHz CPU with 8 GB RAM.

### 6.1. BCI Competition III Dataset IVa

Dataset IVa is recorded from five healthy subjects, labeled as “aa,” “al,” “av,” “aw,” and “ay,” with visual cues indicated for 3.5 s performing right hand and foot motor imagery. The EEG signal has 118 channels and markers that indicate the time points of 280 cues for each subject, band-pass filtered between 0.05 and 200 Hz, and downsampled to 100 Hz.

Before feature extracting for comparison experiment, the raw data needs some preprocessing. Firstly, we extract a time segment located from 0.5 to 3 s and employ FastICA to remove artifacts arising from eye and muscle movements. Secondly, we chose 21 channels over the motor cortex (CP6, CP4, CP2, C6, C4, C2, FC6, FC4, FC2, CPZ, CZ, FCZ, CP1, CP3, CP5, C1, C3, C5, FC1, FC3, and FC5) that related to motor imagery.

In order to improve the performance of CSP and CSSP, we apply Butterworth filter for EEG signals filtering within a specific frequency band between 8 and 30 Hz, which encompasses both the alpha rhythm (8–13 Hz) and the beta rhythm (14–30 Hz) that relate to motor imagery. Then, we program MATLAB code to get spatial filter parameters and feature vectors by variance. Finally, a LDA classifier is used to find a separating hyperplane of the feature vectors.

**Figure 1:**
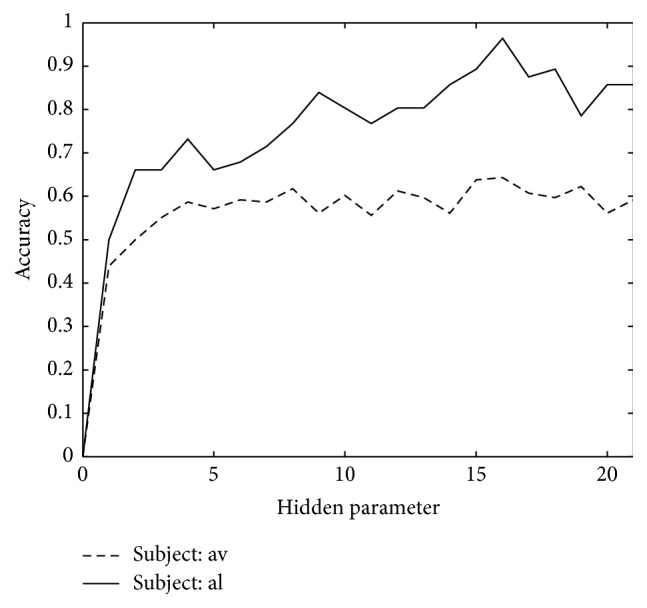
The relationship between hidden parameter and accuracy for LDSs. We choose “al” and “av,” which are the highest and lowest accuracy performance, respectively, to show the relationship between hidden parameter and accuracy.

In LDSs model, the value of a hidden parameter describing dimension of Riemannian feature space is closely related to final accuracy. We chose the highest accuracy performance subject “al” and the lowest accuracy performance subject “av” to show the relationship between hidden parameter and classification accuracy. The result of experiment is presented in Figure 1, which indicates that the accuracy tends to increase when the value of hidden parameter augments approximately and the highest accuracy happens near hidden parameter value of 16.

Then five methods including CSP, CSSP, LDSs, LR+CSP, and LR-LDSs are compared with each other. The results are listed in Table 1.

From Table 1, the bold figures present the best performance results. LR-LDSs are in the majority. The last row shows that the mean of LR-LDS classification accuracy is much better than CSP and a little higher than the others. Comparing with CSP and LR+CSP, LR method is very efficient and useful to improve accuracy. LDSs related methods outperform CSP and CSSP due to their both spatial and temporal features extraction.

### 6.2. BCI Competition IV Database 2a

Database 2a consists of EEG data from 9 subjects. There are four different motor imagery tasks including movement of the left hand, right hand, both feet, and tongue. At the beginning of each trial, a fixation cross and a short acoustic warning tone appear. After two seconds the subject is cued by an arrow pointing to either the left, right, down, or up that denote the movement of left hand, right hand, foot, or tongue for 1.25 s. Then the subjects carry out the motor imagery task for about 3 s. The BCI signals are sampled by 25 channels including 22 EEG channels and 3 EOG channels with 250 Hz and bandpass-filtered between 0.5 Hz and 100 Hz.

Different from dataset IVa, database 2a is a multiclassification problem. However, LDA is a two-class classifier. Therefore, we choose *K*-NN algorithms for CSP, CSSP, LDSs, LR+CSP, and LR-LDSs methods uniformly.  Table 2 describes the classification accuracies results of five above concerned methods. Similar to the results of BCI Competition III Dataset IVa, the mean accuracies of LDSs, LR+CSP, and LR-LDSs are higher than CSP and CSSP methods. Furthermore, LR-LDSs method abstains the best performance.

## 7. Conclusion

CSP has gained much success in the past MI-BCI research. However, it is reported that CSP is only a spatial filter and sensitive to frequency band. It needs prior knowledge to choose channels and frequency bands. Without preprocessing, the result of classification accuracy may be poor. LDSs can overcome these problems by extracting both spatial and temporal features simultaneously to improve the classification performance. Furthermore, we utilize a low-rank matrix decomposition approach to get rid of noise and resting state component in order to improve the robustness of the system. Then LR+CSP and LR-LDSs methods are proposed. Comparison experiments are demonstrated on two datasets. The major contribution of our work is realization of LDSs model and LR algorithm for MI-BCI pattern recognition. The proposed LR-LDSs methods achieve a better performance than CSP and CSSP.

## Supplementary Material

This supplementary material includes three algorithms programed by MATLAB: LDSs function which output two feature matrices, Martin Distance function which describes the distance between two feature space, the classification method of KNN which classifies the different EEG trails.

## Figures and Tables

**Algorithm 1:**
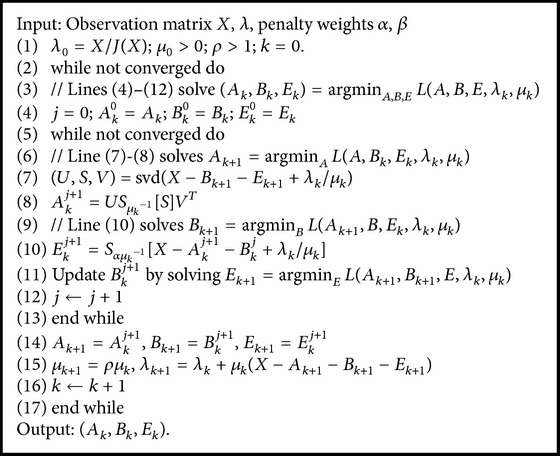
Low-rank decomposition via the inexact ALM method.

**Table 1:** Experimental accuracy results (%) obtained from each subject in BCI Competition III Dataset IVa for CSP, CSSP, and our proposed algorithm (LDS).

Subject	aa	al	av	aw	ay	Mean
CSP	71.43	94.64	61.22	89.28	73.02	77.918
CSSP	77.68	96.43	63.27	**90.63**	79.37	81.476
LDSs	78.57	96.43	**64.29**	90.18	79.76	81.846
LR+CSP	77.68	96.43	63.78	90.18	79.76	81.566
LR-LDSs	**79.46**	**98.21**	63.78	90.18	**80.56**	**82.438**

**Table 2:** Experimental accuracy results (%) obtained from each subject in BCI Competition IV Database 2a for CSP, CSSP LDSs, LR+CSP, and LR-LDSs methods.

Subject	A01E	A02E	A03E	A04E	A05E	A06E	A07E	A08E	A09E	Mean
CSP	90.27	53.13	91.67	71.18	61.11	64.24	79.86	91.32	92.36	77.24
CSSP	90.97	56.94	92.01	72.92	61.81	65.28	79.86	93.06	92.71	78.40
LDSs	91.67	55.56	93.06	74.31	62.50	**70.83**	80.56	93.75	93.06	79.48
LR+CSP	92.01	58.68	**95.14**	74.65	61.81	65.28	**81.25**	94.44	**93.40**	79.63
LR-LDSs	**92.01**	**59.02**	94.44	**75.35**	**63.19**	69.44	**81.25**	**95.14**	93.06	**80.32**
