# Supplementary material for: Low-Rank Linear Dynamical Systems for Motor Imagery EEG
Source: Comput Intell Neurosci. 2016 Dec 21;2016:2637603. doi: 10.1155/2016/2637603 (PMC5210283; doi:10.1155/2016/2637603)
Supplement: Supplementary file 1 — This supplementary material includes three algorithms programed by MATLAB: LDSs function which output two feature matrices, Martin Distance function which describes the distance between two feature space, the classification method of KNN which classifies the different EEG trails. [file 2637603.f1.pdf]

Supplementary Material to Low-rank Linear Dynamical Systems for Motor Imagery EEG

1. Algorithm 1 presents LDSs programed by MATLAB.

This program realizes the LDSs function that describes the feature of EEG signals. When we input EEG data to this program, two matrices are output, which one represents spatial feature and another one denotes temporal feature.

Algorithm 1 (LDSs function)

Input: $Y(t) \in R^m$, EEG signal sample; $h \in R$, hidden parameter; $nor \in R$, iteration coefficient

Output: A $\in R^{n\times n}$, temporal transition matrix; $C \in R^{m\times n}$, spatial transition matrix; $B \in R^{n\times n_v}$, noise component coefficient;

1. *Ymean = mean(Y,2);* % get mean of $Y(t)$
2. *[U,S,V] = svd(Y-Ymean);* % Compute the singular value decomposition for removing mean Y
3. $C = U(:,1:h)$; % C is the first h terms of U
4. *X = S(1:h,1:h)*V(:,1:h)';* % $\hat{X} = \Sigma\ V^T$
5. $A = [X(:,2)X(:,3) \ldots\ X(:,m)] \times pinv[X(:,1)X(:,2) \ldots\ X(:,m-1)]$;
   % pinv is matrix pseudoinverse
6. *A = A/nor;* % normalization and iteration
7. $V = [X(:,2)X(:,3) \ldots\ X(:,m)] - A \times [X(:,1)X(:,2) \ldots\ X(:,m-1)]$; % Compute V
8. *[Uv,Sv,~] = svd(V);* % Compute the singular value decomposition of V
9. *B= Uv(:,1);* % Compute B

---

2. Algorithm 2 is the method of Martin Distance realization.

This program realizes Martin Distance function that describes the distance between two outputs of LDSs from two trails of EEG. If the distance value is small, that means two EEG trails have similar feature and may be same pattern of brain activity.

Algorithm 2 (Martin Distance function)

Input: $A1, C1, A2, C2$ where $A1, A2 \in R^{n\times n}, C1, C2 \in R^{m\times n}$;

Output: d Martin Distance between $(A1, C1)$ and $(A2, C2)$

1. *Oaa= dlyap(A1',C1'*C1);* % Compute O_{aa} and dlyap(·) is the discrete Lyapunov function
   *Oab=dlyap(A1',A2,C1'*C2);* % Compute O_{ab}
   Oba = Oab';
   *Obb = dlyap(A2',C2'*C2);* % Compute O_{bb}
2. *[~,Saa,~] = svd(Oaa);*
   [~,Sab,~] = svd(Oab);
   [~,Sbb,~] = svd(Obb); % Compute eigenvalue
3. $d = -sum(2 * log(diag(\mathrm{Sab}))) + sum(log(diag(\mathrm{Saa}))) + sum(log(diag(\mathrm{Sbb})))$; % Compute Martin Distance

3. **Algorithm 3 is the classification method of KNN by MATLAB.**

This program is KNN classification function that classifies different EEG trails data by comparing Martin Distance. We choose some data as training and label them. After we input testing EEG data and calculate by LDSs and Martin Distance functions, the predicted labels of testing data and accuracy are output.

Algorithm 3 (predict result)

Input: traindata, testdata, trainlabel,testlabel

Output: prelabel, accuracy

1. *train=LDS(traindata);* % Compute train.A, train.C by LDS function
   test=LDS(testdata); % Compute test.A, test.C by LDS function
2. *for i=1:p j=1:q* % p denotes EEG training trails, q is the number of EEG testing trails
   K(i,j) = Martindistance (test.A,test.C,train.A,train.C);
   % Compute Martin distance between different EEG trails
   end
3. *[~,NN] = sort(K,2);* % find Nearest Neighbors, NN is the distance matrix
4. *prelabel= trainlabel(NN(:,1));* % get the predict label
5. *accuracy = sum(trainlabel (NN(:,1))== (testlabel)/size(testlabel,1);*
   % Compute predict accuracy
